# Does the time of day in orthopedic trauma surgery affect mortality and complication rates?

**DOI:** 10.1186/s13037-019-0186-4

**Published:** 2019-02-05

**Authors:** Sascha Halvachizadeh, Henrik Teuber, Paolo Cinelli, Florin Allemann, Hans-Christoph Pape, Valentin Neuhaus

**Affiliations:** Department of Trauma, University Hospital Zurich, University of Zurich, Rämistrasse 100, CH-8091 Zurich, ZH Switzerland

**Keywords:** Time of surgery, Complications, Mortality, Trauma surgery

## Abstract

**Background:**

Orthopedic trauma surgery has multiple, both patient-based and surgeon-based risk factors. Evaluating and modifying certain patient safety factors could mitigate some of these risks. This study investigates the influence that the time of day of surgery has on mortality and complication rates.

**Question/purpose:**

This study evaluates whether the time of day of orthopedic trauma surgery influences complication or mortality rates.

**Patients and methods:**

A prospective Swiss surgical database developed as a nationwide quality assurance project was reviewed retrospectively. All patients with trauma-coded diagnoses that were surgically treated in Swiss hospitals between 2004 and 2014 were evaluated. Surgery times were stratified into morning, afternoon, evening and night. The primary outcomes were in-hospital mortality and complication rates. Co-factors were sought in bivariate and multivariable analysis.

**Results:**

Of 31,692 patients, 13,969 (44.3%) were operated in the morning, 12,696 (40.3%) in the afternoon, 4,331 (13.7%) in the evening, and 550 (1.7%) at night. Mortality rates were significantly higher in nighttime (2.4%, OR 1.26, *p*=0.04) and afternoon surgery (1.7%, OR 1.94, *p*=0.03) vs. surgery in the morning (1.1%). Surgery performed in the afternoon and at night showed significantly increased general complication rates vs. surgery performed in the morning. (OR 1.22, *p*=0.006 and OR 1.51, *p*=0.021, respectively).

**Conclusion:**

This study observed higher complication and mortality rates for surgery performed after-hours, which correlates with other recent studies. Surgeon fatigue is a potential contributing factor for these increased risks. Other potential factors include surgeon experience, surgery type, and the potential for more severe or emergent injuries occurring after-hours.

## Background

Orthopedic trauma surgery can save lives and is usually performed to control acute bleeding and to stabilize major fractures. However, surgical interventions influence mortality and complication rates [1, 2]. Patient-related factors, comorbidities, and injury severity are important risk factors affecting surgical outcomes [3–5]. Perioperative circumstances also impact complication and mortality rates [6–8]. To evaluate patient safety in surgery, recent studies have assessed surgery start time as a potential factor that can affect surgical outcomes [9, 10]. Some authors have speculated that time-related factors play a role in patient safety and have identified seasonal outcome phenomena. For example, Inaba et al. [11] observed increased complication rates in patients treated in July, which corresponds with the beginning of the academic year for new US medical residents. The authors described this finding as the “July Phenomenon.” Further authors have demonstrated increased complication and mortality rates at the beginning of the academic year [12]. Differences in trauma severity and injury distribution are seen during weekends, which may be a result of changes in human behavior [13].

Providing around-the-clock medical care with a constant in-hospital presence of medical specialists creates a significant socio-economic burden and may impact not only patients but also caregivers [14]. Several studies in the fields of internal medicine, intensive care medicine, obstetrics, interventional cardiology, and abdominal surgery have shown potentially higher complication rates in after-hour surgical care [15–24]. Further, medical decision making may be affected at night, as Singh et al. showed increased rates of unknown diagnoses and a trend toward increased false negative appendectomies in the setting of acute appendicitis [25].Orthopedic trauma surgery is also offered 24 h a day. We are unaware of any study evaluating the outcome of orthopedic trauma patients based on the time of the day they underwent surgery. We tested two hypotheses with respect to orthopedic trauma surgery:

1. Does the time of day of surgery have a significant effect on mortality or complication rates in orthopedic trauma patients?
2. Are patient age, ASA-classification, and the surgeon’s experience risk factors for increased mortality or complications after orthopedic trauma surgery?

## Methods

### Study design

For this study a prospective surgical database (Arbeitsgemeinschaft für Qualitätssicherung, AQC) was retrospectively reviewed [26]. Most surgical departments in Switzerland are members of this initiative, as it is an important measure of quality control, surgical education and scientific analysis [27, 28]. Since the establishment of AQC in the mid-90s, more than 70 Swiss surgical departments prospectively document their inpatient surgical cases online via a tool called AdjumedCollect (Adjumed Services AG, Zurich, Switzerland).

The AQC-database is a two-part questionnaire. The first part collects surgical data including date, time, type of surgery (elective vs. emergent and inpatient vs. outpatient), Swiss surgical procedure classification (CHOP) codes [29], the surgeons experience level (resident, fellow, attending or chief of surgery), and intra- or postoperative complications. Surgery start time is an optional field. The second part of the questionnaire collects patient data including age, gender, diagnosis codes [30] according to ICD-10 [31], the ASA (American Society of Anesthesiologists) physical status classification [32], length of stay, ICU admission, general complications, and need for antibiotic treatment.

### Definitions

The AQC database distinguishes between three forms of inpatient surgical complications:

- A: General complications such as pulmonary, cardiovascular, gastrointestinal, renal, or neurological complications.
- B: Intraoperative complications such as iatrogenic fractures or nerve, tendon, or vascular damage
- C: Postoperative complications such as bleeding, infection, impaired wound healing, or incorrect fracture reduction (axial, rotational or length)

Since this study exclusively evaluated the most common orthopedic trauma surgeries, we included only orthopedic related intra- und postoperative complications. An overview of those complications that were analyzed are listed in the Addendum. Mortality was defined as in-hospital mortality.

### Study subjects

The inclusion criteria were all patients admitted to a Swiss hospital between 2004 and 2014 with an ICD-10 diagnosis code starting with an S- or T- code, which therefore includes injuries, intoxication and other external sequelae. Patients were excluded if the start time of surgery or other relevant data were missing.

### Variables and outcome measures

For bio-statistical reasons we categorized the day into four segments as follows: “group AM” for surgeries starting between 7:00 AM and 12:59 PM; “group PM” for surgeries starting in the afternoon between 1:00 PM and 6:59 PM; “group EV” for surgeries starting in the evening between 7:00 PM and 11:59 PM; and “group N” for night-time surgeries between midnight and 6:59 AM.

In-hospital mortality was the primary outcome measure. Secondary outcome measures were overall, intra- and postoperative complication rates during the primary hospitalization.

The following confounders were assessed: age, gender, ASA classification, type of surgery (emergent or elective), experience of the operating surgeon, duration of surgery in minutes and duration of hospitalization in days.

It is important to note that all Swiss residents are required to have general health insurance. About 25% of Swiss residents may, however, choose semi- or full private health insurance which offers higher comfort during hospitalization and the option to be directly treated by the department chair or a senior attending surgeon. This factor may be a relevant confounding factor.

### Statistical analysis

Data was extracted online through the Adjumed Analyze evaluation tool (Adjumed Services AG, Zurich, Switzerland) and analyzed using SPSS Statistics program (Version 23, IBM software, Armonk, New York, United States) as well as GraphPad Prism (Version 7.00 for Windows, GraphPad Software, La Jolla California USA, www.graphpad.com).

Statistical analyses were performed with unpaired t-tests and Pearson’s chi-squared test. Mean and standard deviations were expressed as continuous data, frequencies and percentage as dichotomous data. Variables were sought in bi- and multivariate analysis (binary logistic regression analysis). Time of surgery was the explanatory variable in the statistical models, while other factors were added in a backward likelihood option. A *p*-value of < 0.05 was considered statistically significant.

## Results

### Demographic data

**Fig. 1:**
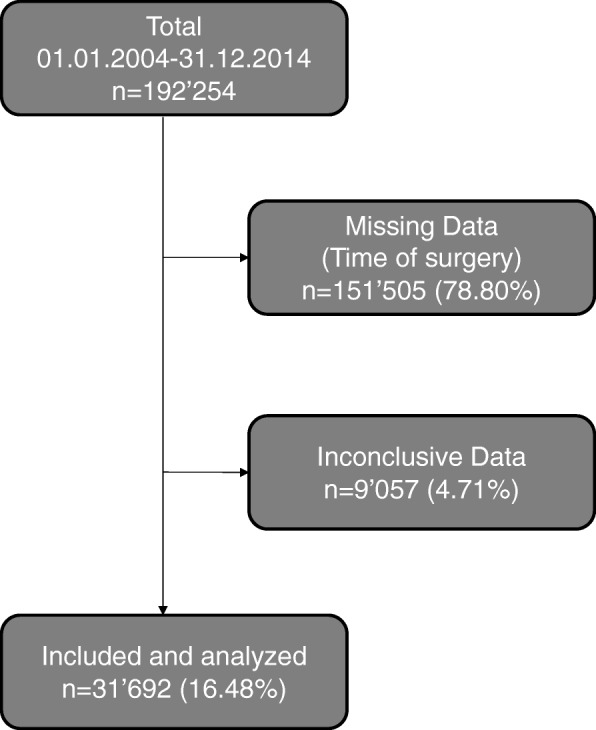
Flowchart diagram of included and analyzed data. The 16.48% of included cases represent high quality data since they were entered precisely and complete

This study included 192,254 patients with S- or T-coded diagnoses that were admitted to Swiss hospitals between 01.01.2004 and 31.12.2014. After excluding 151,505 (78.80%) cases due to missing time of surgery data and 9057 (4.71%) cases with otherwise missing data, a total of 31,692 (16.48%) cases were analyzed (Fig. 1). The study cases were mainly open or closed reduction and internal fixation of radius and ankle fractures, total or partial hip arthroplasty in the setting of femoral neck fractures, but also arthroscopic or soft tissue surgeries (Table 1). We consider the dataset in this study to be representative and of high quality, since the data in the AQC database is entered by the operating physicians, invested in providing accurate surgical data, themselves.

**Table 1:**
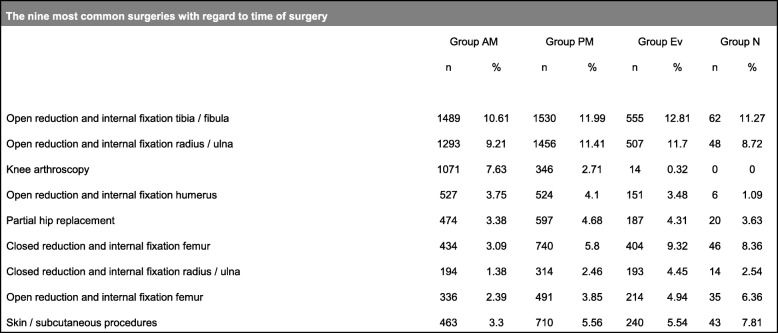
The nine most common surgeries with regard to time of surgery

### Timing of surgery

**Fig. 2:**
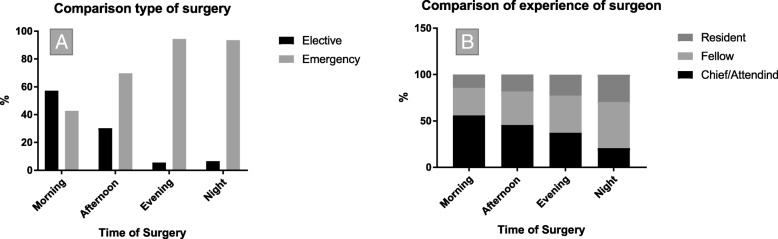
**a** Distribution of type of surgery depending on the daytime the procedure was started. **b** Surgeons with most of experience (chief surgeons of attendings) performed mostly during the day, whereas midlevel experienced (fellows and residents) performed more in the evening and at night

Surgery occurred in the morning in 44% of cases (group AM), while 40% occurred in the afternoon (group PM), 14% in the evening (group EV), and the remaining 1.7% of procedures were performed during the night (group N). Overall, 61.7% of surgeries were emergent and 38.3% were elective. The most common surgeries that were performed in groups EV and N were emergency surgeries, while 57.2% of surgeries occurring in the morning were elective (Fig. 2, a). More experienced surgeons (department chair or senior attendings) performed 48.6% of all cases, with 55.9% of their cases occurring in the morning, whereas midlevel surgeons or residents performed 79.3% of nighttime surgeries (Fig. 2, b).

### Mortality

**Fig. 3:**
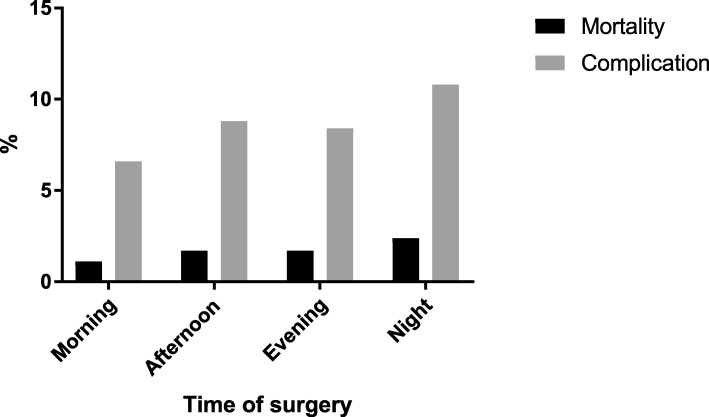
The overall complication rate, as well as the mortality rate rise during the day and are highest after surgeries are performed at night

The overall mortality rate was 1.4%, ranging from 1.1% in morning surgeries (group AM) and 2.4% in nighttime surgeries (group N) (Table 2). In multivariable analysis, surgeries performed in the afternoon or night had a statistically significant increased mortality rate vs. surgeries performed in the morning (OR 1.26, 95% CI: 1.01–1.59, *p* = 0.04 and OR 1.94, 95% CI: 1.07–3.53, *p* = 0.03 respectively). Further, age, male gender, ASA III and IV classification, and emergency surgery were predictors of mortality with statistically significant increased odds ratios (Table 3). ASA III and IV classification were most strongly associated with an increased risk of mortality with an OR of 2.28 (95% CI: 1.63–3.21, *p* = < 0.001) and 12.89 (95% CI: 8.45–19.69, *p* = < 0.001) respectively. Figure 3 summarizes mortality rates and the overall complication rates for the different time groups.

**Table 2:**
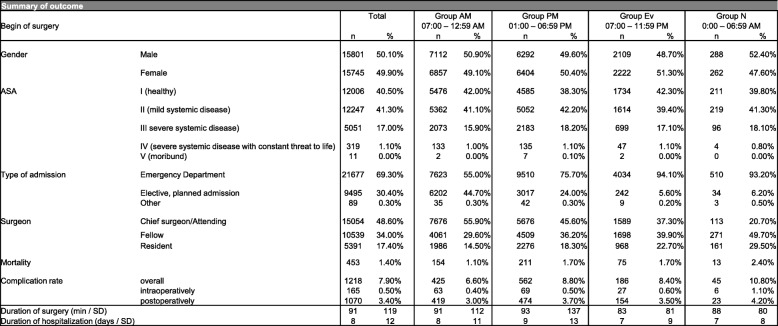
Summary of outcome stratified into time of day surgery was performed

N (number), ASA (American Society of Anesthesiologists), SD (Standard deviation)

**Table 3:**
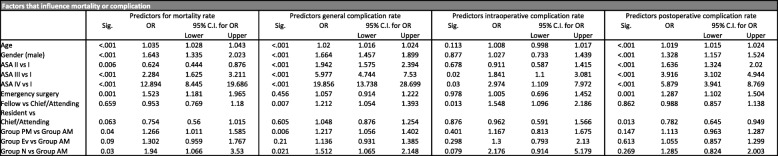
Comparison of factors that influence mortality or complication rate

Sig (Significance), OR (Odds Ratio) CI (Confidence interval)

### Complications

The overall complication rate was 7.9%, ranging from 6.6% in group AM and 10.8% in group N (Fig. 3). The overall postoperative surgical complication rate was 3.4% and the rate was highest in nighttime procedures at 4.2% (Table 2). Intraoperative complications were documented in 0.5% of all cases and the rate was twice as high in nighttime procedures at 1.1%.

In multivariable analysis, the time of day of surgery was not associated with increased intra- or postoperative surgical complications (Table 3). However, the intraoperative complication rate during nighttime procedures vs. morning procedures trended towards significance with an OR of 2.18 (95% CI: 0.91–5.18, *p* = 0.079).

General complication rates were significantly increased between patients who received surgery in the afternoon (group PM) and at night (group N) vs. those patients who had surgery in the morning (group AM) with odds ratios of 1.22 (95% CI: 1.06–1.40, *p* = 0.006) and 1.51 (95% CI: 1.07–2.15, *p* = 0.021) respectively. Additionally, age, male gender and ASA II, III and IV classification were significant predictors for general and postoperative complications (Table 3). ASA III and IV classification as well as surgeon experience (OR 1.55, 95% CI: 1.10–2.19, *p* = 0.013) were the only significant predictors for intraoperative complications, while emergent surgery was an additional risk factor for postoperative complications (Table 3).

## Discussion

Medical caregivers strive to provide best possible care, while maximizing safety to do no harm. However, complications and death are part of medicine, and especially in orthopedic trauma. This study analyzed in-hospital complication and mortality rates in patients after orthopedic trauma surgery. We analyzed all orthopedic trauma cases with complete data in the prospective AQC database and compared outcomes based on the starting time of surgery. Of the 30′000 plus orthopedic surgical cases, surgeries performed at night were mostly emergency cases whereas approximately 60% of surgeries that began in the morning were elective and were predominantly performed by more experienced surgeons. Emergency surgeries and surgeries performed in the afternoon or at night were significant predictors of mortality.

These results correlate with a trend reported by Chacko et al. [23]. They compared two groups of similar patients who needed hip surgery. The first group (*n* = 499) had surgery between 7 AM and 6 PM, the second group (*n* = 268) between 6 PM and 7 AM. Early mortality was 7.4% in the daytime group compared to 9.3% in the nighttime group. The results are also comparable to those of Yount et al. [33], who compared cardiac surgery starting before or after 3 PM. They showed a doubling in absolute and risk-adjusted mortality in late cardiac surgery. The total costs of hospitalization were 8% higher in the late surgery patient cohort. However, late cases were more urgent than early cases.

Higher ASA classification as well as older age and emergency operations were further predictors of mortality. Previous studies showed similar results [34–37]. Since elective surgery is seldomly performed at night, there is little data assessing whether the safety of nighttime surgery itself or rather the emergent situation of an acutely injured patient has a greater impact on mortality rate. Possible nighttime risk factors for mortality include staffing constraints. For example, Wallace et al. [38] showed reduced mortality in patients treated at low-intensity intensive care units where an in-house intensivist was present at night.

This study analyzed intraoperative, postoperative and general complication rates. Our data found no significant differences in intra- or postoperative complication rates between after hour and daytime surgery. However, general complication rates were significantly higher when surgery started in the afternoon or at night.

This correlated with a study from Ricci, Leighton and colleagues [22] which prospectively investigated surgical outcomes of femoral and tibial shaft fractures and compared daytime (6 AM to 4 PM) to after-hours (4 PM to 6 AM) surgery. They compared complication rates of 70 patients who underwent surgery during the day and 82 patients who underwent surgery at night. After hour surgeries were predictors for higher general complication rates, reoperation and hardware removal rates [22]. While the focus was on comparing immediate versus delayed treatment, a higher rate of technical complications leading to revision was found in the after-hours group. For example, the nighttime group had a twofold higher likelihood of undergoing an unplanned reoperation (34 vs. 17%).

Possible causes of increased mortality and complications are the higher rate of emergent surgeries with acute injuries that require surgery or higher injury severity as seen in multiple injured patients [39]. Our data supports this since only general complication rates were significantly increased in after-hours surgery. Further, there were higher rates of ASA III and lower rates of ASA I classified patients operated after-hours (Table 2). Additionally, surgeon fatigue is an important potential safety factor. For example, Taffinder et al. [19] found that sleep-deprived surgeons committed 20% more errors and took 14% longer during a laparoscopy simulator exercise. Having an orthopedic surgical specialist in-house after-hours may be one method of increasing patient safety, as shown by lower peri-operative complication rates in patients operated by in-house vs. on-call surgeons in a study from Earley et al. [40].

### Strengths and limitations

This study has several strengths as well as limitations. Being a retrospective analysis, no functional outcome measures were obtained with this large sample size. The outcome measures were based on absolute numbers of complications, intraoperative parameters and mortality. Complications and mortality could be underreported if patients were transferred to other hospitals for postoperative care. In this study with a large dataset, an argument could be made for big data bias potential, such as multiple comparisons bias, [41] but these results correlate well with our experience in a level 1 trauma center. Overall, very few patients (1.7%) were operated at night. This comparably small sample size may affect the analysis and increases the chance for statistical error. The AQC database provides anonymous data, that prevents controlling or adding missing data such as specific scoring systems. Physicians enter the data manually, which suggests proper and accurate documentation and allows us to dependably analyze the data. An important consideration is that this database does not document injury severity or other risk factors thereby preventing a risk-adjustment analysis. However, because common surgeries were performed, and based on the overall complication and mortality rates, one could deduce that the included cases were neither patients suffering life-threatening injuries nor severely injured patients. Finally, the AQC database records only in-hospital data and cannot assess long-term outcomes.

## Conclusion

Orthopedic trauma surgery performed in the afternoon or at night had significantly higher mortality rates as well as general, but not intra- or postoperative complication rates vs. orthopedic trauma surgery performed in the morning. This may be a result of higher rates of acute emergencies that require immediate surgery, higher injury severity, as well as surgeon factors, such as fatigue, after-hours. It is critical to maximize patient safety and provide the best possible care for patients at any time of the day. This includes surgeon self-awareness and carefully choosing when to operate. Finally, further study may be necessary to help identify modifiable risk-factors in after-hours surgery.

## Abbreviations

AM: Ante meridiem
AQC: Arbeitsgemeinschaft für Qualitätssicherung in der Chirurgie (Task force for quality assurance in surgery)
ASA: American Society of Anesthesiologists
CHOP: Schweizer Operationsklassifikation (Swiss surgery classification)
CI: Confidence Interval
Ev: Evening
ICD: International classification of disease
ICU: Intensive care unit
N: Night
OR: Odds Ratio
PM: Post meridiem
